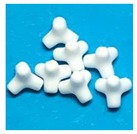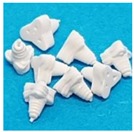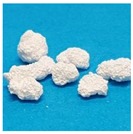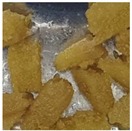# Calcium Phosphate Bone Graft Substitutes with High Mechanical Load Capacity and High Degree of Interconnecting Porosity

**DOI:** 10.3390/ma12213471

**Published:** 2019-10-23

**Authors:** Georg Hettich, Ronja A. Schierjott, Matthias Epple, Uwe Gbureck, Sascha Heinemann, Hadi Mozaffari-Jovein, Thomas M. Grupp

**Affiliations:** 1 Aesculap AG, Research & Development, Am Aesculap-Platz, 78532 Tuttlingen, Germany; ronja_alissa.schierjott@aesculap.de (R.A.S.); thomas.grupp@aesculap.de (T.M.G.); 2 Department of Orthopaedics, Physical Medicine and Rehabilitation, Department of the Ludwig-Maximilians-Universität München Marchioninistrasse 15, 81377 Munich, Germany; 3 Inorganic Chemistry and Center for Nanointegration Duisburg-Essen (CeNIDE), University of Duisburg-Essen, Universitaetsstr. 5-7, 45117 Essen, Germany; matthias.epple@uni-due.de; 4 Department for Functional Materials in Medicine and Dentistry, University of Wuerzburg, Pleicherwall 2, 97070 Wuerzburg, Germany; uwe.gbureck@fmz.uni-wuerzburg.de; 5 INNOTERE biomaterial, Meissner Str. 191, 01445 Radebeul, Germany; sascha.heinemann@innotere.de; 6 Institute of Materials Science and Engineering Tuttlingen (IWAT), Furtwangen University, Kronenstraße 16, 78532 Tuttlingen, Germany; Hadi.MozaffariJovein@hs-furtwangen.de

**Keywords:** calcium phosphate, granules, biomechanics, bone graft substitutes

## Abstract

Bone graft substitutes in orthopedic applications have to fulfill various demanding requirements. Most calcium phosphate (CaP) bone graft substitutes are highly porous to achieve bone regeneration, but typically lack mechanical stability. This study presents a novel approach, in which a scaffold structure with appropriate properties for bone regeneration emerges from the space between specifically shaped granules. The granule types were tetrapods (TEPO) and pyramids (PYRA), which were compared to porous CaP granules (CALC) and morselized bone chips (BC). Bulk materials of the granules were mechanically loaded with a peak pressure of 4 MP; i.e., comparable to the load occurring behind an acetabular cup. Mechanical loading reduced the volume of CALC and BC considerably (89% and 85%, respectively), indicating a collapse of the macroporous structure. Volumes of TEPO and PYRA remained almost constant (94% and 98%, respectively). After loading, the porosity was highest for BC (46%), lowest for CALC (25%) and comparable for TEPO and PYRA (37%). The pore spaces of TEPO and PYRA were highly interconnected in a way that a virtual object with a diameter of 150 µm could access 34% of the TEPO volume and 36% of the PYRA volume. This study shows that a bulk of dense CaP granules in form of tetrapods and pyramids can create a scaffold structure with load capacities suitable for the regeneration of an acetabular bone defect.

## 1. Introduction

Materials for bone defect treatment in orthopedic applications have to fulfill various demanding requirements. In most cases the materials are subjected to a considerable mechanical load. Therefore materials with high mechanical load capacities are required, such as metallic biomaterials, including titanium or tantalum in the form of porous implants [1,2,3] or high strength acrylic bone cement polymethylmethacrylate (PMMA) [4,5]. Since these materials are not degradable in vivo, only a replacement of the anatomical function is achieved rather than a regeneration of the bone or the joint. Furthermore, due to a stress-shielding effect related to the high stiffness of the aforementioned materials, additional bone in the periphery of these materials may be lost [6,7]. When the implant has to be revised, the size of the bone defect further increases and the fixation of the revision implant subsequently becomes more difficult [8,9]. Decreasing the extent of the bone defect is crucial to obtaining better implant survival rates in revision joint replacement [10,11].

Common techniques to achieve the regeneration of bone defects involve the transplantation of autografts or allografts, as well as the implantation of synthetic bone graft substitutes predominantly based on calcium phosphate (CaP) [12,13]. CaP bone graft substitutes include grafts obtained by the (hydro)thermal processing of native bone [14] and fully synthetic CaP, such as hydroxyapatite (HA) or tricalcium phosphates (TCP) [15]. The bone graft substitutes are usually applied in form of putties [16], granules [17], blocks [18], or self-setting calcium phosphate cements [19]. The material design is predominantly focused on fast tissue ingrowth and bone regeneration rather than on mechanical aspects, since the application site is either mechanically unloaded or additional mechanical support is provided; for example, by plate osteosynthesis techniques [20,21]. Hence, such materials are usually highly porous with an intrinsic microporosity and macropores for a fast ingrowth of bone forming osteoblasts and material resorbing osteoclasts. A minimum macropore size of approximately 100 µm is required [22,23,24,25], whereby a structure with macropores and interconnections larger than 300 µm is recommended for enhanced new bone formation and the invasion of capillaries [15,26]. The beneficial effect of such porous scaffold structures on tissue ingrowth and bone regeneration has been demonstrated in numerous in vitro and in vivo studies [27,28,29,30,31,32]. However, these highly porous bone graft substitutes are unsuitable for a mechanically-loaded orthopedic application, due to the well-known detrimental effect of porosity on mechanical integrity [33].

Previous approaches to increase the mechanical capacity of CaP were mainly focused on the development of composites with an additional ductile and/or degradable phase [34]. This was demonstrated by the fabrication of TCP–iron composites by either mechanical alloying [35] or plasma-spark sintering [36], as well as by a combination with polymers, such as polylactic-co-glycolic acid or polycaprolactone [37]. Although these approaches were successful to a certain extent at achieving improved mechanical properties, they were associated with other complications, such as the formation of acidic (polymers) or particulate (iron oxide) degradation products with potential inflammation reactions at the implantation sites.

Therefore, the objectives of this study were to develop a novel approach of a “pure” CaP bone graft substitute with load capacities and to compare this approach with commonly used materials for bone defect filling in orthopedic applications. Dense granules in specific shapes were used as bulk material in a way that (a) granules have contact to one another in order to provide mechanical load capacity of the bulk material, and (b) the space between the granules is retained in order to provide an appropriate scaffold structure for bone regeneration. It was hypothesized that with this novel approach, the contradiction between porosity and mechanical integrity of CaP scaffolds could be solved such that sufficient mechanical load capacity and scaffold structure (porosity, pore sizes, and interconnectivity) are achieved. Two different granule designs, i.e., tetrapods and pyramids, were chosen and fabricated either by powder injection molding techniques (tetrapods) or by additive manufacturing techniques (pyramids). Tetrapods were made of β-tricalcium phosphate (β-TCP) and pyramids of a biphasic material consisted of HA and β-TCP. The intra-granular space, i.e., the space within the granule material itself, was minimized by an applied sintering regime, leading to mostly dense ceramics with only marginal microporosity. Different granule designs, fabrication techniques, and CaP formulations were chosen to consider different variations of the approach.

Commercially available, porous CaP granules and morselized bone chips were used as reference materials. These reference materials represented two commonly used orthopedic treatment options for bone defect regeneration. The commercial CaP granules are used as filling material for revision hip surgery; e.g., for the reconstruction of the acetabulum and to fill the shaft [38]. Morselized bone chips are used within the impaction bone grafting technique in revision joint replacements [39,40,41,42].

These four materials were analyzed by physico-chemical (scanning electron microscopy and X-ray diffraction), mechanical (impactions and cyclic compressions), and micro-computer tomography (micro-CT) techniques. The obtained data were evaluated with a special focus on the mechanical stability (i.e., changes in bulk height) and the scaffold structure after mechanical loading (i.e., bulk porosity, pore size and interconnectivity).

## 2. Materials and Methods

### 2.1. Material Synthesis and Granule Fabrication

Four different bulk materials were tested in this study (Table 1). Shapes of tetrapods (TEPO) and pyramids (PYRA) were inspired by previous studies on specifically-shaped granular bone graft substitutes [43,44]. Screening tests revealed that the TEPO and PYRA shapes provide an optimal compromise between mechanical stability and bulk porosity. More hollow shapes (e.g., hollow tetrahedrons) provided higher porosity but not enough mechanical stability, whereas more spherical shapes (e.g., cylinders) provided higher mechanical stability but not enough bulk porosity.

Tetrapods with an edge length of 3.3 mm were fabricated by the Fraunhofer Institute for Manufacturing Technology and Advanced Materials (IFAM, Bremen, Germany) using powder injection molding (BoyXS, Dr. BOY GmbH and Co. KG, Neustadt-Fernthal, Germany). Powder injection molding allowed the usage of multiple tetrapod cavities in one mold, increasing the economic efficiency of the manufacturing process. A commercially available β-TCP powder (Plasma Biotal, Derbyshire, UK) and a polyethylene-based binder were used as feedstock. TCP is an inherently soluble CaP with a higher solubility than HA. The β-phase is thermodynamically stable up to 1125 °C, while above this temperature the α-phase occurs [45]. Debinding of the green parts was performed by ethanol washing and sintering in air. The sintered specimens had a relative density of approximately 95% of the theoretical density of 3.07 g/cm^3^. The overall crystallinity was about 93%, leaving a small amorphous share.

Pyramids with an edge length of 3.5 mm were fabricated by INNOTERE (INNOTERE biomaterial, Radebeul, Germany) using extrusion-based 3D printing at room temperature, followed by carrying out the cement reaction and washing. The pyramids were manufactured using a specific CaP paste and a custom-made printing protocol [46]. The density resulted from the material density of the cement paste and was about 2 g/cm^3^. The shape was determined by the printing process on a flat surface, where the contour tapered from the flat bottom to top without undercuts. This allowed high throughput using a multichannel printer and high density. The green parts were sintered afterwards such that a biphasic material consisting of HA and β-TCP resulted.

Commercially available, porous CaP granules (CALC; Calcibon; Zimmer Biomet, Warsaw, IN, USA) with a size between 3 and 4 mm consisted of nanocrystalline HA and CaHPO_4_ (monetite) were used as the first reference material. Morselized, cancellous bone chips (BC) with an edge length between 5 and 8 mm were prepared from three fresh frozen human femoral heads and were used as the second reference material. The usage of human bone was approved by the Ethics Committee of the University of Heidelberg (S-170/2016). According to the recommended clinical preparation procedure, the femoral heads were first sawed into slices and the cortical bone was removed before the spongy bone was manually morselized using a bone nibbler [41,47].

### 2.2. Material Characterization Techniques

Scanning electron microscopy (SEM) of CaP granules prior to mechanical loading was performed with an ESEM Quanta 400 instrument (ThermorFisher Scientific, Waltham, MA, USA) with gold/palladium-sputtered samples. SEM of bone chips was performed with a Zeiss Evo 50 instrument with gold sputtered samples. X-ray powder diffraction (XRD) was performed on a Bruker D8 Advance diffractometer (Bruker, Billerica, MA, USA) with Cu Kα radiation (1.54184 Å) in Bragg–Brentano geometry on thoroughly ground sample on a silicon sample holder. The software TOPAS 4.2 (Bruker, Billerica, MA, USA) was used for quantitative XRD analysis by Rietveld refinement analysis.

### 2.3. Mechanical Analysis

**Figure 1:**
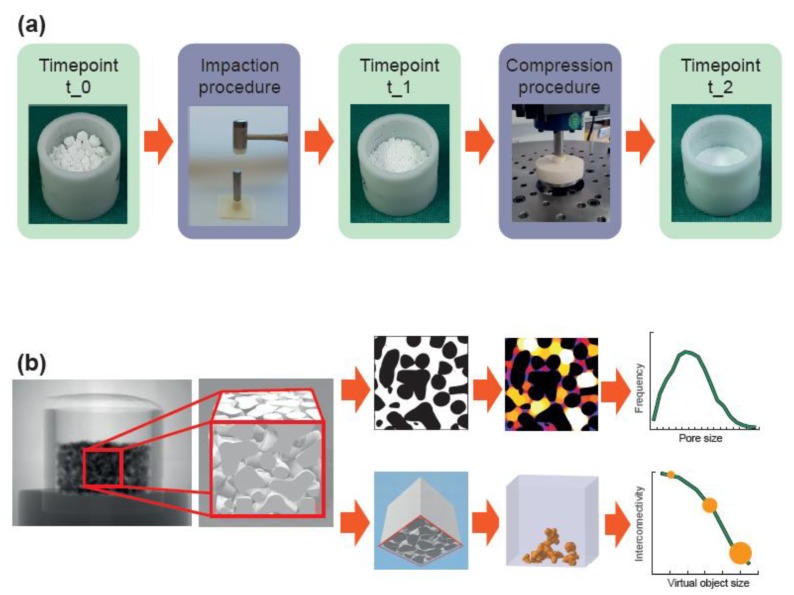
Workflow of the mechanical and morphological analyses. (**a**) The material, loosely filled in a container (timepoint t_0), was loaded according to the impaction bone grafting technique (impaction procedure). After the impaction procedure (timepoint t_1), the material was compressed by applying the estimated loads behind an acetabular cup (compression procedure) resulting in a realistic load scenario (timepoint t_2). (**b**) A cube with a 10 mm edge length in the center of the container was analyzed with a micro-CT scanner. In a first analysis, dense material (shown in black) was separated from the pore space (shown in white/colored) to obtain the porosity, the average pore size, and the pore size distribution. In a second analysis, dense material was also separated from pore space but the cube was additionally “closed” on five sides. By simulating the penetration of a virtual object with various diameters into the porous system from the remaining “open” side, the interconnectivity in terms of accessible volume (shown in orange) with respect to the virtual object size could be obtained.

The mechanical analysis was designed to approximate the mechanical load that would occur when the material is applied to reconstruct an acetabular bone defect during hip joint replacement. Two different mechanical loads were applied (impaction and compression procedure) and three different timepoints (t_0, t_1, and t_2) were considered (Figure 1a). The impaction procedure was designed to simulate the load occurring during the impaction bone grafting technique. The compression procedure was designed to simulate the load behind an acetabular cup occurring during daily activities after patient remobilization. Timepoint t_0 represents the situation where the material is filled in the defect without any load, timepoint t_1 represents the situation after the impaction procedure, and timepoint t_2 represents the situation after impaction and compression procedure.

The materials were filled in a cylindrical container with a diameter of 23 mm and filling heights of 14 mm for granules and 30 mm for bone chips. These heights were chosen such that a height of approximately 13 mm resulted after the impaction and compression procedures. For the impaction procedure, the containers were placed on a rubber pad, a metallic punch was placed on the materials (22 mm diameter, 75 mm height), and ten impactions with an orthopedic hammer (30 cm, 410 g) were applied [48,49,50]. For the compression procedure, a spherical punch with a diameter of 22 mm was used to apply a load with a peak force of 1.52 kN (corresponding to a peak pressure of 4 MPa) in as sinusoidal waveform with a frequency of 3 Hz. The chosen peak pressure of 4 MPa corresponds to the estimated contact pressure of acetabular cartilage and the loads around acetabular cups [51,52,53,54,55]. Screening tests revealed a settling behavior of the materials before 100,000 load cycles, such that a number of cycles was limited to 100,000. The compression procedure was conducted using a resonance pulsator (Newline 10 kN, PneuSys software, SincoTec, Clausthal-Zellerfeld, Germany).

Six filled containers of each bulk material were tested. The height of each bulk material was measured at each timepoint. The heights were normalized to that at t_1 at 100% in order to compare the decrease in height between the materials from timepoint t_1 to t_2, which might be an indicator for primary stability of the implant.

### 2.4. Morphological Analysis

Morphological analysis (Figure 1b) was performed using a micro-CT system (phoenix v|tome|x, General Electric, Boston, MA, USA). In total, eight scans per material were performed. For each bulk material, a scan of one representative container at t_0 and t_1 and scans of all six containers at t_2 were conducted. The containers were fixed in the micro-CT scanner by a custom-made device so that the containers rotated exactly around their vertical axes. Images were taken at 120 kV, 140 mA, and 0.25° rotation steps. The focus was adjusted to cover a 15 mm × 15 mm × 15 mm cube in the center of the container. 1000 images with 1000 × 1000 pixels were captured, resulting in a final voxel size of 15 µm.

Image processing was performed with the software ImageJ (Version 1.51k, National Institutes of Health, Bethesda, MD, USA). Segmentation of dense material (i.e., CaP and trabecular bone) from the pore system (i.e., inter-granular pores, intra-granular pores and trabecular spacing) was performed with manually-defined global density thresholds. Inaccuracies at the image boundaries were eliminated by cutting the image stack into 667 images with 667 × 667 pixels in the center of the container, resulting in a “region of interest” (ROI) of 10 mm × 10 mm × 10 mm. A median filter with the radius of three pixels was applied to reduce image noise. Porosity, pore size, and pore size distribution were analyzed with the BoneJ functions “Volume Fraction,” “Thickness,” and “Histogram” in ImageJ [56,57]. Porosity represents the volume fraction of pore volume with respect to the total volume in percentage. Pore size represents the diameter of the greatest sphere that fits within the pore space. Pore size distribution represents the frequency of voxels dedicated to specific pore sizes. This is displayed as a histogram in which the dedicated voxels were summarized in 100 µm intervals and plotted relative to all pore voxels in percentages. Interconnectivity in terms of accessible volume was defined as the volume accessible for a virtual object which penetrates the scaffold from one side, a method called “directional shrink-wrap” [58,59]. It was calculated using the function “ROI Shrink-Wrap” in the CTAn software (Version 1.18, Bruker, Kontich, Belgium). For different object diameters, the interconnectivity was defined as the ratio of accessible volume with respect to the total volume in percent. The virtual object diameter was chosen as 10 voxels, 30 voxels, and 50 voxels, representing diameters of 150 µm, 450 µm, and 750 µm. Five surfaces of the ROI were closed such that only one surface was accessible for the virtual object. The accessible surface was chosen to be at the opposite side of the force application, since this side would be directed towards the native bone, and hence represents the side for potential bone ingrowth (Figure 1b).

### 2.5. Statistical Analysis

All data results are presented as means ± standard deviations. Heights are shown at all timepoints, whereas porosity, pore size, pore size distribution, and interconnectivity are only shown at timepoint t_2. Statistical differences between the materials were analyzed by a Mann–Whitney U test with a level of significance of *p* < 0.05 using the function ranksum in Matlab (The Mathworks, Natick, MA, USA).

## 3. Results

### 3.1. Material Characterization Results

**Figure 2:**
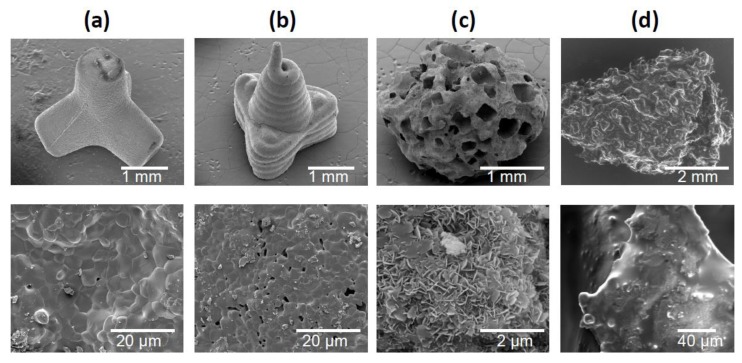
Scanning electron micrographs of granules and bone chips used in this study. (**a**) Tetrapods, (**b**) pyramids, (**c**) Calcibon granules, and (**d**) morselized bone chips.

Figure 2 shows SEM images of the three granule types and the bone chips. TEPO showed a slide edge, as expected for the powder injection fabrication (Figure 2a). In contrast, PYRA showed signs of the extrusion-based molding process where the pasty starting material was applied. At higher magnification, both objects showed primary crystals after sintering with a diameter of a few µm. In contrast, CALC showed an irregular porous structure wherein the rectangular/cubic pores were obviously created by a leaching process. At higher magnification, primary crystallites in the sub-µm range indicated a recrystallization in the presence of water. BC showed the irregular shape and the open-porous structure of trabecular bone.

**Figure 3:**
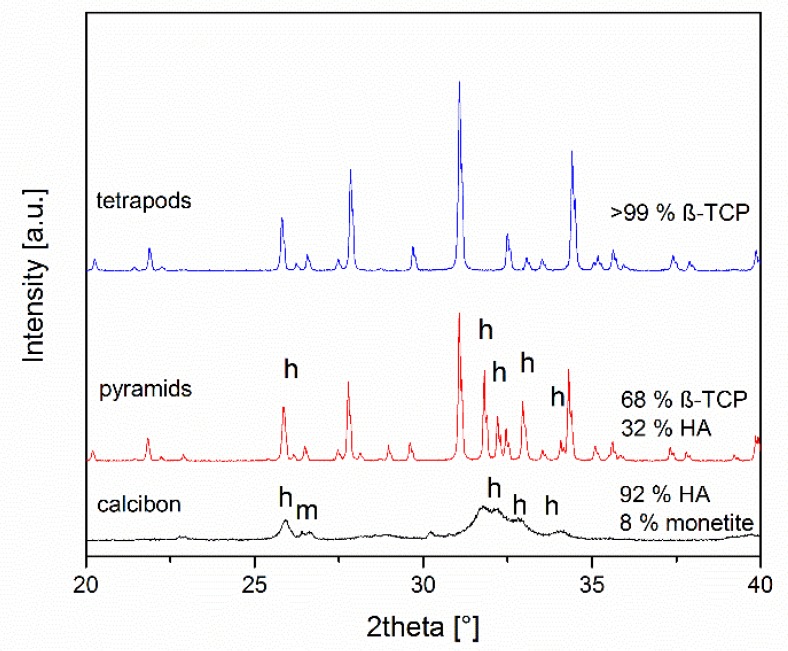
X-ray diffraction patterns and quantitative phase composition according to Rietveld refinement analysis of the granules. All peaks for tetrapods correspond to ß-tricalcium phosphate, the other peaks result from hydroxyapatite (h) and monetite (m).

The compositions of the objects prepared were assessed by X-ray powder diffraction after grinding (Figure 3). TEPO consisted of phase-pure β-TCP. In contrast, PYRA contained both β-TCP and hydroxyapatite in a weight ratio of about 68:32. CALC consisted of approximately 92 wt% hydroxyapatite and 8 wt% monetite. Bone chips consist mainly of collagen and nanoscopic calcium phosphate particles [12]. In the powder diffractogram of bone chips, only calcium phosphate particles are visible (data not shown) [60].

### 3.2. Mechanical Results

**Figure 4:**
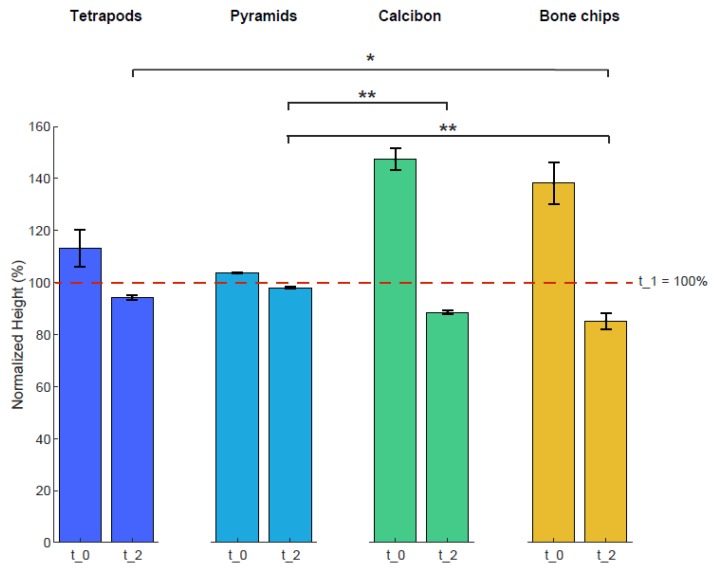
Heights of the four bulk materials before and after mechanical loading. The height of each sample was normalized to that of timepoint t_1 at 100%. Shown are mean and standard deviation values at t_0 and t_2. Statistically significant differences between the materials are shown for timepoint t_2 with ∗ = *p* < 0.05 and ∗∗ = *p* < 0.01.

Heights of the four bulk materials within the containers were used to characterize mechanical stability after mechanical loading (Figure 4). The height of each sample was normalized to the height of t_1 with 100%. The averaged normalized height of TEPO at t_0 was 113% ± 8% and decreased to 94% ± 1% at t_2; that of PYRA was 104% ± 1% and decreased to 98 ± 1%; that of CALC at t_0 was 147% ± 4% and decreased to 89% ± 1%; and that of BC was 138% ± 8% and decreased to 85% ± 3%. Statistically significant differences at timepoint t_2 were observed between tetrapods and bone chips (*p* < 0.05), between pyramids and Calcibon (*p* < 0.01), and between pyramids and bone chips (*p* < 0.01).

### 3.3. Morphological Results

**Figure 5:**
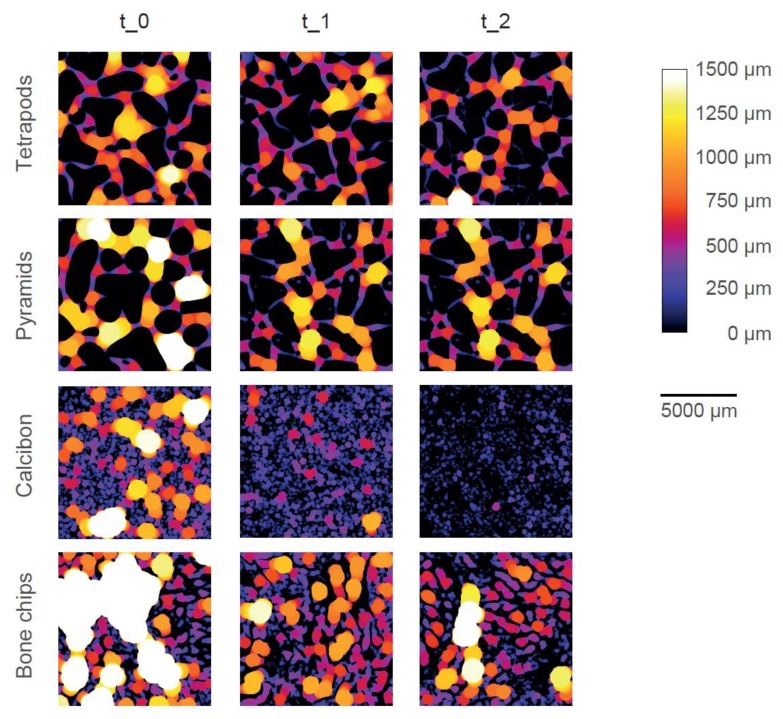
Graphical representation of the pore space of one typical specimen of each bulk material at each timepoint. The size of the pore space is color-coded according to the color bar. Dense material is shown in black. With increasing pore size, the brightness increases, until pore size above 1500 µm is shown in white.

Color-coded micro-CT images show the pore system of one typical example of each bulk material at each timepoint (Figure 5). Dense material is shown in black (i.e., CaP or trabecular bone) and pores are color-coded according to their size and the given color bar. TEPO and PYRA only showed inter-granular pores (i.e., pores between the granules) and no intra-granular pores (i.e., pores within the granules themselves). TEPO and PYRA remained mainly stable during the impaction and compression procedures such that only few fragments were clogging the pore space. Therefore, inter-granular pore space only slightly decreased due to mechanical loading. In contrast, CALC showed inter-granular pores and intra-granular pores at t_0. After the impaction procedure (t_1), inter-granular pores diminished due to granule crushing and only intra-granular pores remained. After the compression procedure (t_2), a complete granule disruption led to an almost dense material without any pores. BC showed inter-granular pores and intra-granular pores at t_0 and intra-granular pores at t_1. Since the trabecular bone structure mainly withstood the mechanical loading, BC was the only material which showed intra-granular pores after the mechanical loadings.

**Figure 6:**
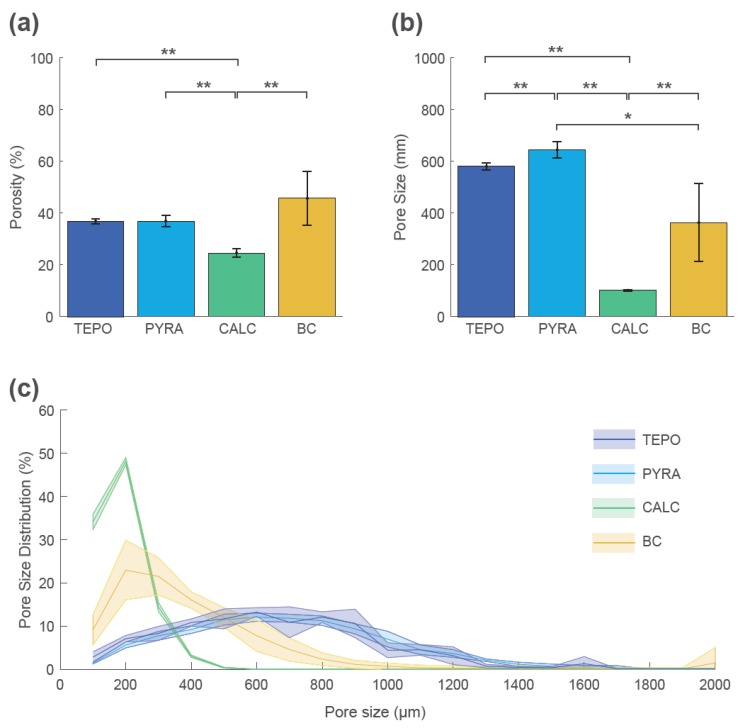
Porosity, pore size, and pore size distribution of tetrapods (TEPO), pyramids (PYRA), Calcibon granules (CALC), and bone chips (BC) at timepoint t_2. (**a**) Porosities and (**b**) pore sizes as mean and standard deviation values; statistically significant differences are indicated as ∗ = *p* < 0.05 and ∗∗ = *p* < 0.01. (**c**) Pore size distribution histogram summarized in 100 µm steps as mean values (solid lines) and standard deviations (shaded areas).

These qualitative findings were confirmed by quantitative analyses at timepoint t_2 (Figure 6). The mean porosity at t_2 was highest for BC (46% ± 10%), lowest for CALC (25% ± 2%), and similar for TEPO and PYRA (37% ± 1% and 37% ± 2%, respectively) (Figure 6a). The mean pore size was highest for TEPO (581 ± 15 µm) and PYRA (640 ± 30 µm), whereas the mean pore size was lower for BC (360 ± 140 µm) and CALC (141 ± 4 µm) (Figure 6b). The pore size distributions are shown as histograms summarized in 100 µm intervals (Figure 6c). The pore space of CALC could be attributed to pore sizes below 500 µm and the pore space of BC to pore sizes below 800 µm. For both materials, most pores were around 200 µm. In contrast, the pores of TEPO and PYRA were more uniformly distributed and could be attributed to pore sizes between 400 µm and 1000 µm.

**Figure 7:**
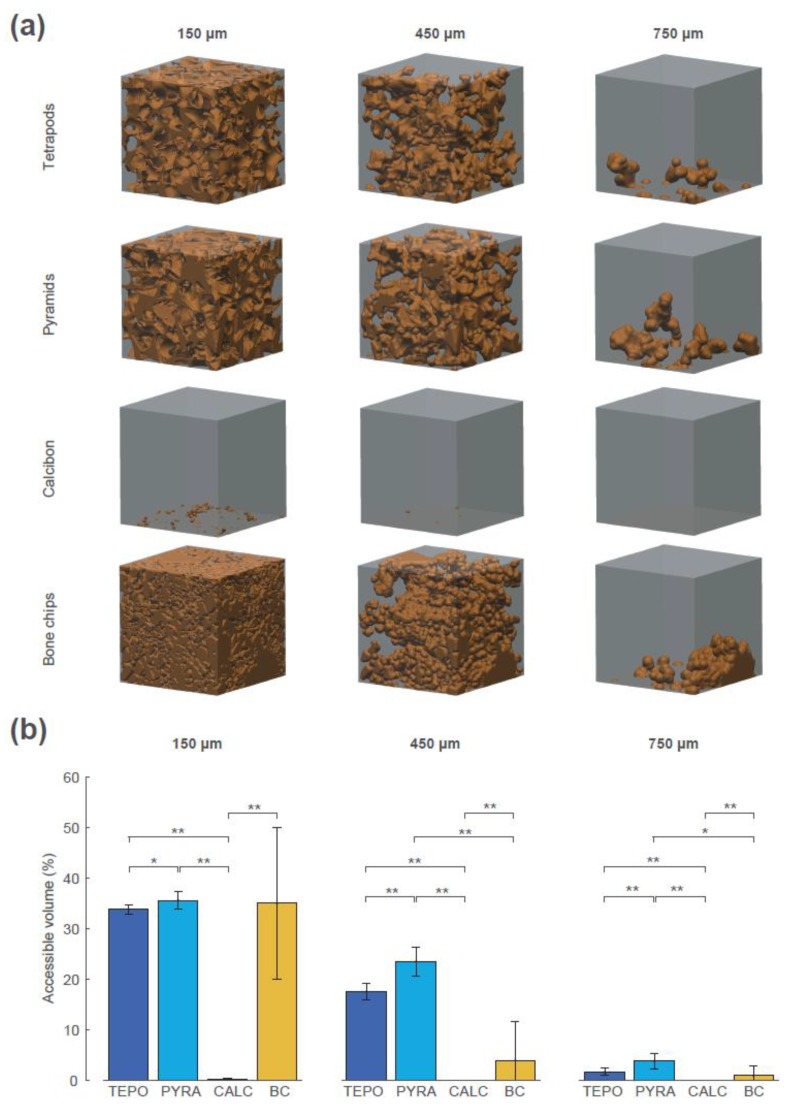
Interconnectivity in terms of accessible volume of the four materials at timepoint t_2 for the virtual object diameters of 150 µm, 450 µm, and 750 µm. (**a**) The accessible volumes of the four materials and the virtual object diameters are graphically shown as brown structures within the regions of interest (ROIs). (**b**) The accessible volumes of the four materials and the virtual object diameters as the ratio between accessible volume, with respect to overall volume in percentage; statistically significant differences are indicated as ∗ = *p* < 0.05 and ∗∗ = *p* < 0.01.

The interconnectivity in terms of accessible volume was assessed using the “directional shrink-wrap” transport pathway analysis (see Methods and Figure 7). The accessible volumes for the virtual object diameters of 150 µm, 450 µm, and 750 µm revealed large differences between the four materials (accessible volume is shown as brown structure within the ROI). A virtual object with a diameter of 150 µm could access 34% of the overall volume of TEPO, 36% of PYRA, 0.1% of CALC, and 35% of BC. The values of TEPO and PYRA are close to their porosity value of 37%, indicating an open-porous system with interconnections larger than 150 µm in both materials. When the virtual object diameter was increased to a diameter of 450 µm, a considerable amount of the TEPO and PYRA volumes were still accessible (18% and 23%, respectively), whereas the accessible volume of BC decreased to 4% and that of CALC completely diminished. With a further increase of virtual object diameter to 750 µm, the accessible volumes of TEPO, PYRA, and BC were reduced to 2%, 4%, and 1%, respectively.

## 4. Discussion and Conclusions

The objectives of this study were to develop a novel approach for CaP bone graft substitutes with sufficient load capacity for orthopedic applications and to compare this approach with commonly used bone grafts and a bone graft substitute. The comparison was based on physicochemical (scanning electron microscopy and X-ray diffraction), mechanical (impaction and cyclic compression), and morphological (micro-computer tomography) methods. The physicochemical characteristics were analyzed as surface topology and phase composition; mechanical characteristics as decreases in height at three timepoints; and morphological characteristics as porosity, pore size, pore size distribution, and interconnectivity. Pore size and interconnectivity are independent measures of scaffold structure [58], whereby pore size defines the diameter of the greatest sphere that fits within the pore space, and interconnectivity defines the volume accessible for a virtual object with a certain diameter.

Most of the commonly used porous CaP bone graft substitutes are inherently brittle, which leads to insufficient mechanical load capacities for orthopedic applications. Much effort has been made to improve mechanical load capacities using different reinforcement strategies [15,61,62]. The strength deficit of synthetic CaP scaffolds compared to human bone is mainly attributed the composite nature of bone and the hierarchical bone architecture from nano-scale to macro-scale, which is very difficult to replicate in a synthetic material [63,64,65]. Another important drawback of CaP scaffolds is that the progress of an initiated crack will not be hindered by the deformation of the material ahead of the crack (as would be the case in a ductile material), causing a complete disruption of the scaffold [15].

In the approach presented here, a bulk of tetrapods or pyramids created a network of interconnected macropores by their inter-granular space, whereby the absent microporosity (intra-granular space) enhanced the overall mechanical load capacity. The approach bypasses the above-described problem of the inherent brittleness of bioceramic scaffolds in the way that a crack of one granule would not lead to a catastrophic failure of the whole structure. It was shown that this scaffold structure is able to withstand mechanical load in an impaction and compression model, suggesting its viability in application, as an acetabular bone-defect-filling material. The load capacity could be seen by the almost constant height of tetrapods and pyramids from timepoint t_1 (100%) to timepoint t_2 (94% and 98% respectively; Figure 4). At the same time, the network of interconnected macropores with pore sizes and interconnections larger than 450 µm remained intact, demonstrating the potential for vascularization and bone remodeling, as proposed by several authors [15,26,31]. This was not the case for commercially available, porous CaP granules, which collapsed under mechanical load, leading to diminished porosity, pore size, and interconnectivity. Although bone chips showed a higher total porosity compared to tetrapods or pyramids after dynamic loading (46% versus 37%), they had smaller mean pore sizes and interconnectivity values for virtual object diameters above 450 µm. Even more important was the decrease in height from t_1 (100%) to t_2 (85%) of BC, which might be a contributing factor to the reduced survival rate of bone chips when applied in large or loaded acetabular bone defects [47,66]. Changes in the bulk heights of tetrapods and pyramids after mechanical loading could be mainly attributed to changes in granule morphology (i.e., a few distal parts of the granules were broken, so the granules moved closer together) and not on the granule material itself. In contrast, changes in bulk heights of porous CaP and bone chips could be attributed to changes in morphology and material (i.e., also the intra-granular pore space decrease under mechanical loading).

Previous studies which already addressed the use of specifically-shaped CaP granules for bone defect filling were mainly focused on scaffold structures rather than on mechanical aspects. These granules were either not intended for load-bearing applications [44] or failed at a compressive load of approximately 3–4 N for a single granule [43]. Studies considering mechanical aspects also compared stacks of macroporous β-TCP granules with different porosities [22,67]. It was shown that a stack of the high porosity granules resembles trabecular bone in its porous microarchitecture [67] and that granules with 4 mm edge length could be used for impaction grafting technique [22]. However, in both studies a single load of 3–5 MPa led to a large compression displacement. A CaP bone graft substitute of dense granules which combines a mechanical load capacity up to 4 MPa with appropriate scaffold structure for bone ingrowth (porosity of 37%; accessible volumes for 150 µm for objects of 34% and 36%, and for a 450 µm objects, 18% and 23%) is novel to the best of our knowledge.

This study was focused on mechanical and morphological aspects and did not include biological considerations. Even though the sintered materials consist of established β-TCP and HA, whose biocompatibility was proven in numerous in vitro, in vivo and clinical studies [65,68], the next step should include a basic cell-culture investigation (e.g., using MTT-assay). Furthermore, the approach should be tested in a more advanced in vitro test setup with a realistic bone model and in combination with the intended prosthesis and direction of load. A clinical application of the approach definitely requires in vivo studies in large animal models with sufficient bone defect size and mechanical loading. Such studies should not only consider the primary stability in vivo, but must also address the change of mechanical stability due to bone tissue ingrowth and subsequent granule degradation.

## Figures and Tables

**Table 1:** Overview of the materials tested.

	TEPO	PYRA	CALC	BC
**Shape**	tetrapods	pyramids	granules	bone chips
**Manufacturing**	powder injection molding	extrusion-based, cement reaction	cement reaction	bone nibbler
**Size**	4–5 mm	3–4 mm	3–4 mm	5–8 mm
**Material**	ß-TCP	68% HA; 32% ß-TCP	nano-HA; Monetite	HA